# CSF lymphocytic pleocytosis does not predict a less favourable long-term prognosis in MS

**DOI:** 10.1007/s00415-022-11521-0

**Published:** 2022-12-24

**Authors:** Lauren Astbury, Seema Kalra, Radu Tanasescu, Cris S. Constantinescu

**Affiliations:** 1 Academic Unit of Mental Health and Clinical Neuroscience, Section of Clinical Neurology, University of Nottingham, Nottingham Centre for MS and Neuroinflammation, Nottingham University Hospitals QMC, Nottingham, NG7 2UH UK; 2 University Hospitals of North Midlands NHS Trust, Royal Stoke MS Centre of Excellence, Stoke On Trent, UK; 3 Cooper University Hospital, Cooper Neurological Institute, Cooper Medical School at Rowan University, Camden, NJ 08103 USA

**Keywords:** Multiple sclerosis, Cerebrospinal fluid, Pleocytosis, Prognosis

## Abstract

**Objective:**

The role of CSF lymphocytic pleocytosis in predicting the clinical outcome of multiple sclerosis is unclear. We explored the impact of CSF pleocytosis at diagnosis on long-term disease progression in a large UK cohort.

**Methods:**

We extracted demographic, clinical and CSF data of people with MS attending the MS clinics between 1996 and 2014 at two MS centres from the English Midlands. We compared EDSS at onset, follow up EDSS and progression indices Multiple Sclerosis Severity Score (MSSS), annualized change in EDSS and transition to secondary progression in the presence/absence of pleocytosis. Two-tailed student *t*-test, Mann–Whitney *U* test, Chi-Square or Fisher’s exact tests were used for detecting the differences.

**Results:**

A total of 247 patients with MS (178 females; mean age 42.4; 217 with relapsing onset) were followed up for an average of 13.56 years (median 12 years). Almost 18% had lymphocytic CSF ≥ 5 per microliter. CSF pleocytosis was not associated with higher EDSS at the time of LP or at follow up, and other progression indices like MSSS, annualized change in EDSS or transition to secondary progression.

**Discussion:**

CSF pleocytosis at MS diagnosis does not predict higher long-term disability and has no long-term prognostic value in routine clinical circumstances. Differences between MS populations and potential differences in disease activity at the time of CSF analysis may account for differences between studies.

**Supplementary Information:**

The online version contains supplementary material available at 10.1007/s00415-022-11521-0.

## Introduction

Multiple sclerosis (MS) is an immunologically mediated disease of the central nervous system (CNS), characterised pathologically by inflammation with damage and loss of myelin and neurons. The neurological deficits follow relapsing–remitting or progressive patterns and lead to accumulating disability [2]. MS has a remarkable clinical variability. Despite important progress made in MS treatments and monitoring, the accurate prediction of long-term outcome at the individual patient level is not yet possible.

The need for easy-to-process, clinically relevant, and routinely available disease biomarkers make CSF studies an ideal choice for biomarker development in MS. CSF OCB have been associated with a worse prognosis in terms of higher risk of conversion of clinically isolated syndrome to clinically definite MS or more severe disability [13]. However, OCB are present in the CSF in most people with MS at diagnosis and, once present, remain positive, and therefore, their use as a potential prognostic biomarker is limited.

CSF lymphocytic pleocytosis has received relatively little attention as a prognostic indicator. It is defined as lymphocyte count of ≥ 5 per microliter. Cross-sectional studies in MS show that CSF pleocytosis correlates with active CNS inflammation and more active MS. CSF pleocytosis is seen early after the MS symptom onset [2] and in association with acute MRI lesions as evident by MS lesions with reduction in apparent diffusion coefficient, suggesting this to be an early and possibly transient phenomenon [3]. Presence of intrathecal pleocytosis is also associated with axonal damage biomarker, serum neurofilament light chain (NfL) in early and active MS [4]. However, there is no correlation of CSF NfL with CSF cell count in progressive MS [9].

CSF cell count can vary between serial analyses in the same individual [1, 14] and despite the variation in CSF count, the ratio between various immune cells remains stable [1]. CSF B cell dominance identified on serial CSF analyses has been linked with more rapid likelihood of progression in MS {Wurth, 2017 #925.

All this suggests that CSF pleocytosis at diagnosis can be a marker of disease activity in early and more active MS, but it is unclear how its presence correlates with disability accumulation beyond the acute phase, and onset and progression of disease over longer time. Lotan and colleagues, in a study in an Israeli MS cohort, showed that intrathecal pleocytosis at diagnosis correlates with more active disease and a worse disease progression after a mean follow-up of 9.4 years [8]. We aimed to assess, in a large UK patient cohort, if CSF pleocytosis at diagnostic lumbar puncture can be a reliable predictor of MS progression.

## Methods

In this retrospective study, we extracted demographic, clinical and CSF data from the MS clinic databases at two large MS centres in the English Midlands: Queen’s Medical Centre in Nottingham and Royal Stoke MS Centre, Stoke-on-Trent. This study received ethical approvals from Nottingham and Staffordshire Research Ethics Committees. The investigators had full access to the patient’s records at their respective centres (LA, RT and CSC-Nottingham, and SK Stoke- on-Trent). All participants gave written informed consent.  The study was performed in accordance to the tenets of the Declaration of Helsinki. We analysed data from consecutive patients who had lumbar puncture as a part of diagnostic work up between 1996 and 2014. These patients were diagnosed using the McDonald criteria 2001 and/or 2010 for those diagnosed after 2010. Patients were categorised into relapse onset MS (RMS) or primary progressive MS (PPMS). Relapsing onset category includes RRMS, CIS (Clinically Isolated Syndrome) and people who later converted to Secondary Progressive MS (SPMS). We recorded the EDSS at onset as First ever EDSS (at diagnosis confirmation), and the latest EDSS score as follow-up EDSS. Follow-up EDSS was also used to calculate the Multiple Sclerosis Severity Score (MSSS) [11]. MSSS provides a progression index, taking EDSS and time to reach the EDSS into account. MSSS scores are in decimal values, with higher values indicating a higher progression index i.e. faster disability accumulation. Additionally, we also used annualized change in EDSS as used by Lotan et al. [8]. We also looked at the proportion of participants who developed secondary progression after 10 years disease duration. CSF Pleocytosis was defined as CSF lymphocyte count ≥ 5 per microliter.

### Statistical analysis

Data were collected at the two centres and then anonymised and amalgamated for analysis. GraphPad Prism^®^ 6 software was used to analyse the data. Two-tailed student *t*-test, Mann–Whitney *U* test or Chi-Square or Fisher’s exact tests were used to compare the 2 groups. The significance level for all analysis is *p* ≤ 0.05.

## Results

### Patient characteristics

A total of 247 MS patients (178 females and 69 males) were included in the study. Around 88% had relapsing onset MS (171 RRMS, 11 CIS, 35 SPMS), and 13% had PPMS (30 PPMS). Average age at the time of MS diagnosis was 39.17 years with a range of 13 to 70 years. CSF lymphocyte counts were taken at diagnosis. The median count was 1.0.

Around 18% (45/247) of the patients showed pleocytosis in CSF with lymphocyte count ≥ 5 per microliter in CSF performed at the time of diagnosis. The median EDSS score at the time of the lumbar puncture was 2.0. The patients were followed up for a mean follow-up duration of 13.56 years (median 12 years). These included 197 patients with follow-up duration between 10 and 30 years (mean 14.84 years) and 50 patients with 6–10 years duration (mean 8.52 years). Follow up median EDSS score was 5.0. Table 1 presents the clinical characteristics of patients in the study.

**Table 1:** Clinical characteristics: demographics, disease characteristics and CSF findings at the time of first LP

Diagnosis	All MS	Relapse onset	PPMS	*p* value
N	247 (RRMS = 182; SPMS = 35; PPMS = 30)	217 (RRMS = 182; SPMS = 35)	30	NA
Sex (% female)	72%	75%	47%	0.01
Age at MS diagnosis (mean; years)	39.17	37.11	54.07	< 0.001
CSF white cell count (mean per microliter of CSF)	3.02	2.81	2.82	NA
CSF Pleocytosis (%)	18% (45/247)	18% (40/217)	16.7% (5/30)	1.000
OCB (% positive-unmatched bands/typical pattern)	91% (116/127)	91% (103/112)	86% (13/15)	1.000
Follow-up time (mean; years)	13.56	13.36	12.70	NA

Relapse onset MS category includes RRMS, CIS and later converted SPMS patients. CSF Pleocytosis was defined as white cell count with ≥ 5 per microliter. Comparisons of means were made using the Student *t* test; and comparison of proportions using the Fisher exact test

*PPMS* primary progressive MS, *OCB* oligoclonal bands, *NA* not applicable

### CSF pleocytosis, lymphocyte count and the disease course

18% of the total MS cohort had CSF pleocytosis (45/247). Clinical characteristics of patients with and without CSF pleocytosis, defined as CSF-pleocytosis and No-CSF pleocytosis groups hereafter, are given in Table 2.

**Table 2:** Comparison of study groups with and without CSF pleocytosis group

Characteristics	Total cohort = 247			> 10 year f/up (total = 197)		
	No-CSF pleocytosis group	CSF Pleocytosis group	*p* value	No-CSF pleocytosis group	CSF Pleocytosis group	*p* value
Diagnosis	*n = *202	*n = *45	NA	*n = *162	*n = *35	NA
Relapse onset	Relapse onset = 177	Relapse onset = 40		Relapse onset = 141	Relapse onset = 30	
PPMS	PPMS = 25	PPMS = 5		PPMS = 21	PPMS = 5	
Sex (% female)	71% (143/202)	77% (35/45)	0.136	71% (116/162)	71% (25/35)	0.983
Age at MS diagnosis (mean; years)	39.32	37.36	0.293	38.47	37.94	0.806
OCB (% positive-unmatched/typical pattern)	92% (92/102)	96%(24/25)	1.00	90% (91/99)	96% (24/25)	1.00
EDSS at onset (median)	2.0	2.0	0.349	2.0	2.0	0.351
EDSS at follow up (median)	5.5	3.5	0.132	5.5	3.5	0.063
Multiple Sclerosis Severity Score (MSSS)	5.51	4.55	0.081	5.37	4.36	0.052
Annualized change in EDSS as ΔEDSS/yr	0.166	0.107	0.174	0.158	0.093	0.072
SPMS conversion after 10 years of disease duration	20% (36/177)	12% (5/40)	0.376	22% (32/141)	10% (3/30)	0.067

CSF Pleocytosis was defined as white cell count with ≥ 5 per microliter. Relapse onset MS category includes RRMS, CIS and later converted SPMS patients. Two-tailed student *t*-test, Mann–Whitney *U* test, Chi-Square or Fisher’s exact tests were used to compare the 2 groups

*PPMS* primary progressive MS, *OCB* oligoclonal bands, *NA* Not applicable

The two groups were similar in terms of age of onset, gender distribution, MS phenotype i.e. relapse onset versus primary progressive, presence of OCB, and disease duration. They also had similar level of disability at the time of diagnosis of MS as measured by EDSS at onset.

Though the CSF-pleocytosis, as compared to No-CSF pleocytosis group, showed a general tendency towards lower EDSS at follow up in-spite of similar EDSS scores at the onset, lower MSSS scores and lower annualised EDSS and lower chances of conversion to SPMS after 10 years follow up, none of these differences in the progression indices reached statistical significance.

**Fig. 1:**
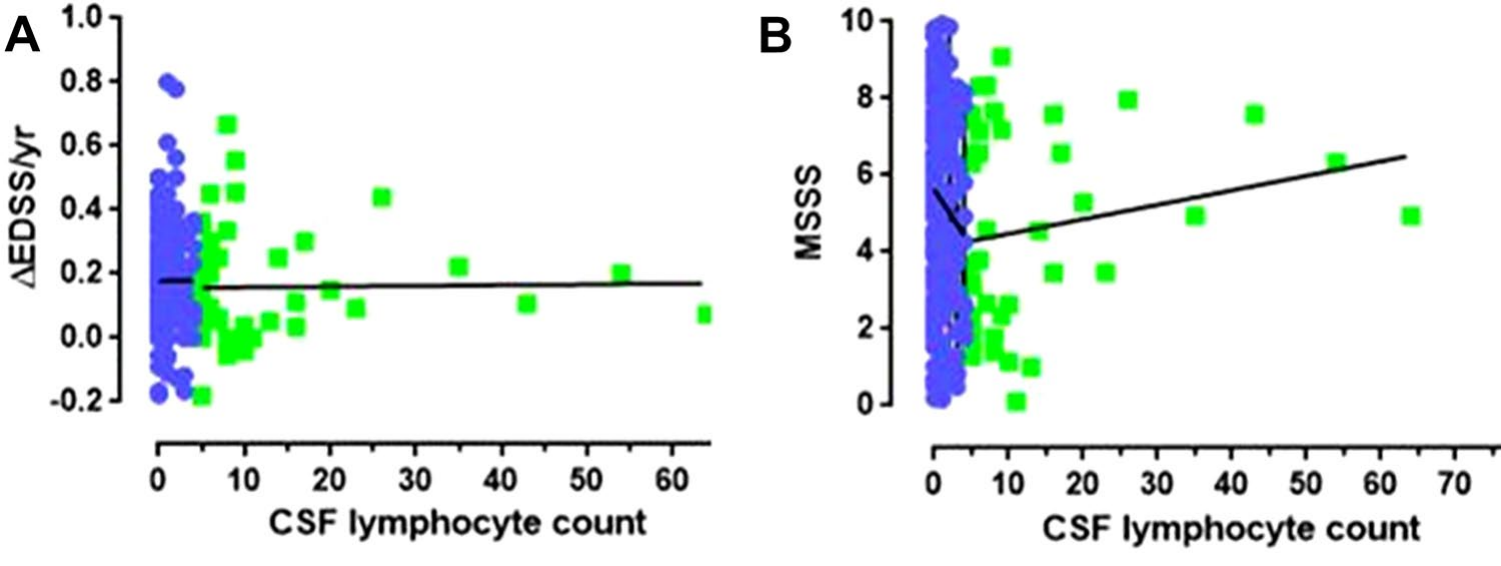
CSF Pleocytosis at the diagnostic lumbar puncture is not associated worse progression indices as seen with higher yearly change in EDSS (annualised change in EDSS = ΔEDSS/yr) (**A**); and Multiple Sclerosis Severity Score (MSSS) (**B**). CSF pleocytosis is represented by green dataset and No-CSF pleocytosis group by blue dataset. None of the correlation coefficients studied were statistically significant. Spearman coefficients for between CSF count and ΔEDSS/yr (*r = *0.082, *p = *0.59); and MSSS (*r = *− 0.16, *p = *0.21) for CSF Pleocytosis group; and CSF count and ΔEDSS/yr (*r = *− 0.026, *p = *0.71); and MSSS (*r = *− 0.11, *p = *0.09) for No-CSF Pleocytosis group

Within the CSF pleocytosis group, no correlation was found between the absolute CSF lymphocyte count and EDSS at onset, EDSS at follow up, MSSS, or annualized change in EDSS. Similar findings were noted for No-CSF pleocytosis group. Figure 1 shows data for correlation between CSF lymphocyte count and annualized change in EDSS (Fig. 1A) and MSSS (Fig. 1B) for CSF- pleocytosis and No-CSF pleocytosis groups.

Oligoclonal bands results were available for a subgroup (*n = *127) out of which 91.3% showed unmatched oligoclonal bands while 8.7% showed atypical results (either no bands (1.5%), or single band, same or overlapping pattern between serum and CSF (7.2%). CSF lymphocyte count was not associated with presence or absence of oligoclonal bands, MS subtype or age at onset of MS.

### Follow up over 10 years

A subset of 197 patients had a follow-up period of ≥ 10 years (median 12 years, range 10–30 years). Table 2 shows characteristics, and comparison with and without CSF pleocytosis within this subgroup.

### Correlation between CSF lymphocyte count and progression indices

We noted no correlation between CSF lymphocyte count and EDSS at onset (*r = *0.017, *p = *0.79); Follow up EDSS (*r = *− 0.03, *p = *0.62). Spearman coefficient for correlation with MSSS was *r = *− 0.155 (*p = *0.06); and for annualized change in EDSS score was *r = *− 0.045 (*p = *0.19) (Supplementary Fig. 1A–D).

Similar to the overall cohort, the sub-group with longer than 10 year follow-up showed no significant correlation between lymphocyte count and EDSS or MSSS values used as progression indices (data not shown).

## Discussion

Our study demonstrates that that CSF pleocytosis at the time of diagnosis in routine clinical circumstances is not a predictor for long-term disability accumulation. This is in contradiction with the results of Lotan et al. [8] who found that high CSF lymphocyte count at diagnosis was associated with worse MS, both in terms of relapses and EDSS scores. The similarities between the two studies are the retrospective design and the comparable length of the mean follow-up (9.4 years for the Israeli cohort, 13.56 years for this study). However, our cohort was larger (*n = *247); more than double of Lotan et al. [8].

The goal of early prediction of outcome in MS is the prevention of accumulating disability. We focused on EDSS and change in EDSS as outcome measure and included MSSS, annualised change in EDSS and proportionate conversion to SPMS as other measures to capture the pace of progression. We did not include the number of relapses in the analysis, as total relapses have no significant effect on reaching high disability levels from disease onset, during the relapsing phase of MS [12]. While Lotan et al. included annual relapse rate, MRI lesion count and electrophysiological findings at the time of presentation in their study, there was no predictive value assigned to these [8].

Lotan et al. [8] included relapse onset patients only and showed a higher proportion of patients with CSF pleocytosis than our cohort (43% versus 18%) but a lower proportion of positivity for oligoclonal bands (64% versus 91% in our cohort). We included progressive onset MS patients. However, analysing only the relapsing MS patients yielded the same results as in total cohort in our study. Our study population was representative of natural history of disease including disease phenotype and oligoclonal band positivity.

While the precise mechanisms driving progression of MS are not fully understood, patient age has impact on disease phenotype. Though papers report immune senescence and decrease in CSF count with age, in normal aging [10] as well as MS [5, 14] the effects are likely to be via particular subtypes e.g. predominantly B cell subtypes [14]. As our cohort was on average only 5 years older at diagnosis than the Israeli cohort, age related explanation is unlikely to explain the difference observed.

Genetic differences may play an important role in explaining the different results. While Israeli patients with MS are similar to the European, including British, populations with respect to the HLA profile that influences the susceptibility to MS, there are population-specific HLA alleles which can influence the clinical course of MS [7].

Our study is a real-world observation of the MS population attending MS clinics. Lumbar puncture and CSF analysis were done as part of the normal diagnostic procedure. The study was conducted over two sites, however the cohorts are geographically close, similar in ethnic makeup, and the sample and data collection were contemporaneous and following similar protocols. Analysing the data separately by centre yielded similar results.

There is paucity of literature addressing this area of research in MS, and there may be a publication bias against studies with “negative findings” [6]. We believe given the accessibility of the CSF cell count and importance of need for biomarkers for progression in MS, our study highlights an important message that similar to a single cross-sectional MRI scan at the diagnosis, CSF pleocytosis detected with routine lumbar puncture at the time of diagnosis is not predictive for long-term disability accumulation.

## Supplementary Information

Below is the link to the electronic supplementary material.

Supplementary file1 Supplementary Figure 1: CSF lymphocyte count and the disease course in complete MS cohort. This figure shows correlation between CSF lymphocyte count and EDSS at onset (A), follow up EDSS (B), Multiple Sclerosis Severity Score (C) and annualized change in EDSS as ΔEDSS/yr (D) in complete cohort of n=247. No significant correlations were identified. Spearman coefficients were used to study correlations. A p-value of <0.05 was considered significant. (JPG 84 KB)

## Data Availability

Primary data may be made available to researcher upon request. Please contact the corresponding author.
