# Supplementary figures and images for: CSF lymphocytic pleocytosis does not predict a less favourable long-term prognosis in MS
Source: J Neurol. 2022 Dec 24;270(4):2042–7. doi: 10.1007/s00415-022-11521-0 (PMC10025177; doi:10.1007/s00415-022-11521-0)

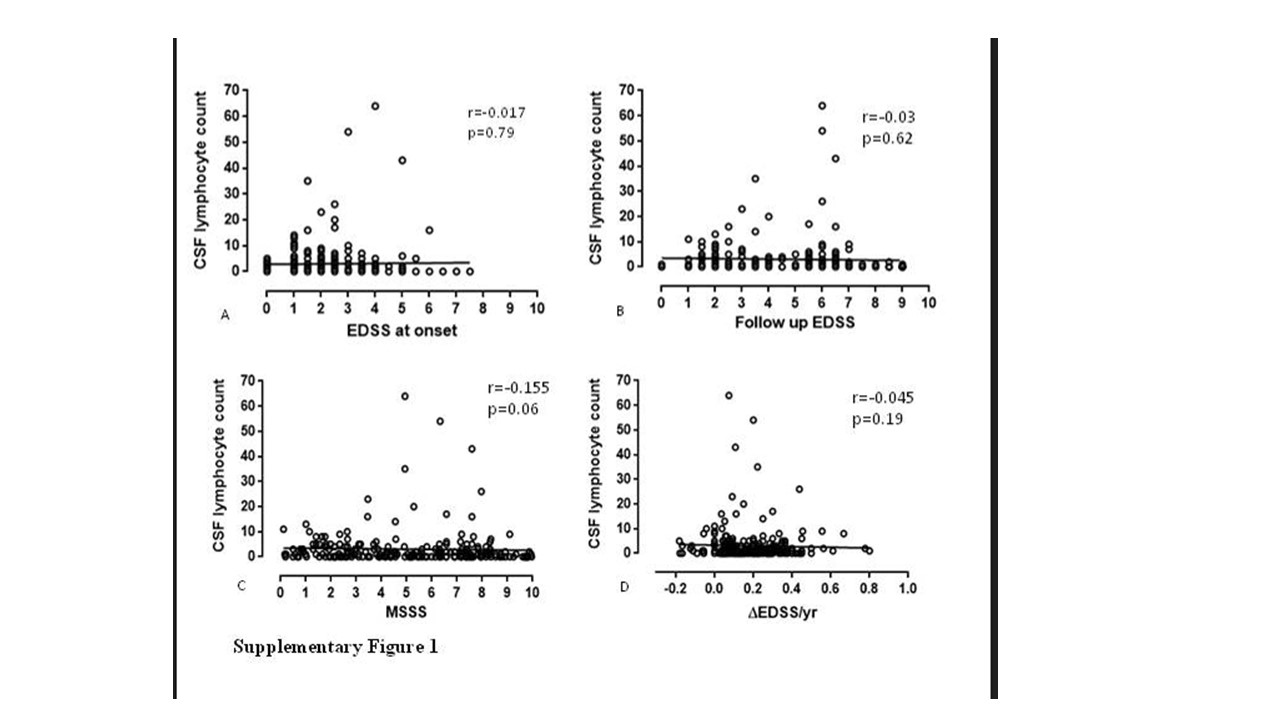

Supplement: Supplementary file 1 — Supplementary file1 Supplementary Figure 1: CSF lymphocyte count and the disease course in complete MS cohort. This figure shows correlation between CSF lymphocyte count and EDSS at onset (A), follow up EDSS (B), Multiple Sclerosis Severity Score (C) and annualized change in EDSS as ΔEDSS/yr (D) in complete cohort of n=247. No significant correlations were identified. Spearman coefficients were used to study correlations. A p-value of <0.05 was considered significant. (JPG 84 KB) [file 415_2022_11521_MOESM1_ESM.jpg]
